# Supplementary material for: Household Fuel Use for Heating and Cooking and Respiratory Health in a Low-Income, South African Coastal Community
Source: Int J Environ Res Public Health. 2019 Feb 14;16(4):550. doi: 10.3390/ijerph16040550 (PMC6406283; doi:10.3390/ijerph16040550)
Supplement: Supplementary file 1 [file ijerph-16-00550-s001.pdf]

Supplementary Materials

Household Fuel Use for Heating and Cooking and Respiratory Health in a Low-Income, South African Coastal Community

Sikhumbuzo Archibald Buthelezi [1,2], Thandi Kapwata [3], Bianca Wernecke [3], Candice Webster [4], Angela Mathee [3,5,6] and Caradee Yael Wright [1,7,*]

1 Department of Geography, Geoinformatics and Meteorology, University of Pretoria, Pretoria 0002, South Africa; archie@mut.ac.za
2 Mangosuthu University of Technology, Durban 4031, South Africa
3 Environment and Health Research Unit, South African Medical Research Council, Johannesburg 2193, South Africa; thandi.kapwata@mrc.ac.za (T.K.); bianca.wernecke@mrc.ac.za (B.W.); angela.mathee@mrc.ac.za (A.M.)
4 University of KwaZulu-Natal, Durban 4041, South Africa; websterc@ukzn.ac.za
5 Environmental Health Department, Faculty of Health Sciences, University of Johannesburg, Johannesburg 2028, South Africa
6 School of Public Health, University of the Witwatersrand, Johannesburg 2000, South Africa
7 Environment and Health Research Unit, South African Medical Research Council, Pretoria 0002, South Africa
* Correspondence: cwright@mrc.ac.za; Tel.: +27-123-398-543

Figure S1. Household and health survey questionnaire.

HOUSEHOLD AND HEALTH SURVEY QUESTIONNAIRE

PLEASE COMPLETE THE FOLLOWING CONTACT DETAILS:

SURNAME: ______________________________

NAME: ______________________________

NODE/ZONE: (Mark the applicable one)

Glebe Node/Zone	1	Ezimbuzini Node/Zone	2	Mangosuthu KwaMnyandu Node/Zone	3

OTHER (SPECIFY) ______________________________

DATE ON WHICH THIS QUESTIONNAIRE IS COMPLETED:

YEAR: ☐☐☐☐ MONTH: ☐☐ DAY: ☐☐

YOUR PERSONAL (RESPONDENT) INFORMATION (Demographic)

1. What is your gender?

a)	Male	1
b)	Female	2

2. What is your date of birth?

YEAR: ____ MONTH: __ DAY: __

3. What is your home language?

a) Afrikaans	1	e)	Zulu	5
b) English	2	f)	Swazi	6
c) Sotho	3	g)	Other	7
d) Xhosa	4			

4. How long have you been living in this town (where you now reside most of the time)?

a)	Since birth	1	d) 4 – 5 years	4
b)	Less than 2 years	2	e) 5 – 10 years	5
c)	2 – 4 years	3	f) More than 10 years	6

5. Where did you live before living in this town?

____

6. How long were you living there?

a)	Since birth	1	d) 4 – 5 years	4
b)	Less than 2 years	2	e) 5 – 10 years	5
c)	2 – 4 years	3	f) More than 10 years	6

THE FOLLOWING QUESTIONS PERTAIN TO YOUR CURRENT RESIDENCE

7. Which of the following best describes your home?

a)	A single family house, not attached to other houses	1
b)	A single family house, attached to other houses	2
c)	A flat	3
d)	Pre-fabricated home (asbestos/wood)	4
e)	A shack	5
f)	Other (define)	

____

8. a) How many rooms are there in the home? (Don't count bathroom and toilet __________

b) How many of these are bedrooms? __________

9. Do you use any of the following heating systems? (Mark Yes **or** No for each one)

a) Wood / coal stove	Yes	No
b) Fireplace	Yes	No
c) Gas heater	Yes	No
d) Asbestos heater	Yes	No
e) Portable electric heater	Yes	No

f) Other (Specify) ______________________________

10. If you do have a portable **gas heater** in your home, how often do you use it during the winter?

a) About every day	1
b) 2 to 3 times a week	2
c) 2 to 3 times a month	3
d) Seldom	4
e) Never	5

11. If you do have a **coal stove** in your home, how often do you use it during the winter?

a) About every day	1
b) 2 to 3 times a week	2
c) 2 to 3 times a month	3
d) Seldom	4
e) Never	5

12. If you do have a **fireplace** in your home, how often do you use it during the winter?

a) About every day	1
b) 2 to 3 times a week	2
c) 2 to 3 times a month	3
d) Seldom	4
e) Never	5

13. What fuel do you **mostly** use for cooking? (Mark only one)

a) Electricity	1
b) Wood	2
c) Gas	3
d) Electricity and gas	4

e) Other (Specify) ______________________

14. Do you use any of the following equipment in your home? (Mark Yes **or** No for each one)

a) Humidifier	Yes	No
b) Air scrubber/cleaner	Yes	No
c) Air conditioner	Yes	No

15. If yes (question 14), how often did you use this equipment the past summer?

a) About every day	1
b) 2 to 3 times a week	2
c) 2 to 3 times a month	3
d) Seldom	4
e) Never	5

16. Where did you use the equipment specified in question 14?

a) All the rooms	1
b) Living room	2
c) Bedroom(s)	3

d) Other (Specify) ______________________

17. Do you open windows or doors to circulate fresh air into your home during the winter months?

a)	Yes	1
b)	No	2

18. How often did you open windows and doors during the previous winter?

a) About every day	1
b) 2 to 3 times a week	2
c) 2 to 3 times a month	3
d) Seldom	4

19. Have you ever had problems with leakages, flooding or water damage in your home?

a)	Yes	1
b)	No	2

20. Have you ever had mold or mildew growing on any surface inside your present home? (e.g. on walls, wallpaper, carpets, ceilings, shower, curtains, etc.)

a) In the shower area (curtain, around bath)	Yes	No
b) In other areas in the home	Yes	No
c) Unknown	Yes	No

21. Which of the following pets do you have? **(Mark more than one if necessary)**

a)	None	Yes	No	e)	Mice	Yes	No
b)	Dog(s)	Yes	No	f)	Bird(s)/Doves	Yes	No
c)	Rabbit(s)	Yes	No	g)	Guinea pig(2)	Yes	No
d)	Cat(s)	Yes	No				

h) Other (Specify) ____

22. Are any of these animals allowed in the home?

a)	Yes	1
b)	No	2

22. Is the home in which you live used on a regular basis by anyone in your household for the following hobbies or crafts? (By regular we mean at least once a week or at least 50 hours per year). **(Mark more than one if necessary)**

a) Pottery and ceramics	Yes	No
b) Painting (Oil)/spray painting	Yes	No
c) Dyes and fibers /fabrics	Yes	No
d) Jewelry and enameling	Yes	No
e) Metal work	Yes	No
f) Automotive (mechanical/electrical)	Yes	No
g) Stained glass soldering	Yes	No
h) Photography (Darkroom use only)	Yes	No
i) Print making	Yes	No
j) Furniture stripping	Yes	No
k) Wood working	Yes	No
l) Silk screening	Yes	No

m) Other, (Specify) ____

INFORMATION ON SMOKING HABITS

23. Have you ever smoked more than one cigarette per day?

a)	Yes (two or more cigarettes/day)	1
b)	No (one or less cigarette/day)	2

24. If yes,

a) Do you currently smoke? a) Yes [1] b) No [2]

b) How old were you when you started smoking? ____________________

c) How many cigarettes do you smoke per day? ______________________

d) If you have quit smoking, how old were you when you quitted? ________________________

25. Is there currently anyone who smokes cigarettes, cigars or pipe on a regular basis (daily) in the home where you live?

a)	No	1
b)	Yes, mother/female guardian	2
c)	Yes, father/male guardian	3
d)	Yes, both parents/guardians	4
e)	Yes, husband/wife	5

f) Yes, other (specify) ________________

26. How many **cigarettes** in total are smoked in the home each day?

a)	None	1	d)	21 – 40	4
b)	1 – 10	2	e)	41 – 60	5
c)	11 – 20	3	f)	More than 60	6

27. How many **cigars** in total are smoked in the home each day?

a)	None	1	c)	6 – 10	3
b)	1 – 5	2	d)	More than 10	4

28. How many times in total is a **pipe** smoked in the home each day?

a)	None	1	c)	6 – 10	3
b)	1 – 5	2	d)	More than 10	4

e) Other substances (specify)

____

GENERAL ACTIVITIES AND LIFESTYLE

29. Do you participate in any of the following activities twice or more per week (Mark Yes **or** No for each one?)

a)	One or more of the following: soccer, rugby, hockey, netball	Yes	No
b)	Aerobics	Yes	No
c)	Exercise with weights	Yes	No
d)	Jogging	Yes	No
e)	Walking	Yes	No
f)	Swimming	Yes	No

h) Other (Specify) ____

EATING HABITS

30. Which of the following do you eat on a regular (at least weekly) basis?

a)	Chicken/Fish	Yes	No
b)	Red meat	Yes	No
c)	Processed food (e.g. polony, meat pies)	Yes	No
d)	Vegetables	Yes	No
e)	Fruit	Yes	No

31. How often do you eat food fried in oil or fat?

a)	Daily	1	b)	Weekly	3
c)	Seldom	2	d)	Never	4

Specify type of oil

____

32. Do you currently consume any form of alcohol?

a)	Yes	1
b)	No	2

33. If yes, how many drinks on average do you consume per week? (One glass of wine, one beer or one tot of spirits is considered as one drink)

a)	Less than 7	1
b)	8 – 14	2
c)	15 – 21	3
d)	More than 21	4

THE FOLLOWING QUESTIONS CONCERN YOUR HEALTH

34. How would you describe your health compared to that of others of the same age group?

a)	Excellent	1
b)	Good	2
c)	Poor	3

35. Do you have any allergies?

a) No - **go to question 42**	1
b) Yes, self-diagnosed	2
c) Yes, diagnosed by a doctor	3
d) Other	4

36. How often do you take medication for the allergy?

a) Never	1
b) Daily	2
c) 2 x a week	3
d) 3 x a week	4
e) Do not know	5

f) Other (Specify) ______________________________

37. What are you allergic to? (Mark each applicable one)

a) Things I eat<br>Specify: ___________________________________	Yes	No
b) Things I inhale<br>Specify: ___________________________________	Yes	No
c) Skin contact<br>Specify: ___________________________________	Yes	No
d) Medication, e.g. Aspirin<br>Specify: ___________________________________	Yes	No

e) Other
Specify: ___________________________________

38. How many **days** over the last **2 weeks** have you been absent from work/studies because of illness?

39. During the **past 12 months**, how many **days** have you been absent from work/study due to illness?

a)	None	1	d)	11 – 15	4
b)	1 – 5	2	e)	16 – 20	5
c)	6 – 10	3	f)	More than 20	6

40. Due mostly to which illness?
Specify ____

41. Have you ever had any of the following illnesses? (Mark No **or** Yes for every illness)

a) Bronchitis	Yes	No
b) Pneumonia	Yes	No
c) Earache	Yes	No
d) Hay fever	Yes	No
e)Chronic bronchitis	Yes	No
f) Sinus trouble	Yes	No
g) Rhinitis	Yes	No
h) Running nose	Yes	No

42. How do you predominantly breathe?
a) Through the mouth 1 b) Through the nose 2

Explain ____

43. When you have a cold, does it normally go to your chest?

a)	Yes	1
b)	No	2

ASTHMA

44. Has your doctor ever said that you have asthma?

a)	Yes	1
b)	No	2

If NO, go to question 54.

45. At what age did your asthma start?

a)	0 – 1 years	1	e)	8 – 9 years	5
b)	2 – 3 years	2	f)	10 years and older	6
c)	4 – 5 years	3	g)	Unknown	7
d)	6 – 7 years	4			

46. a) Do you still have asthma?

a)	Yes	1
b)	No	2
c)	Not sure	3

b) If yes, how frequent

Weekly	1
Monthly	2
Occasionally	3

47. Do you currently take medicine or get treatment for asthma?

a)	Yes	1
b)	No	2

48. If you no longer have asthma, at what age did you last have asthma?

a) 0 - 1 years	1	e) 8 - 9 years	5
b) 2 - 3 years	2	f) 10 years and older	6
c) 4 - 5 years	3	g) Unknown	7
d) 6 - 7 years	4		

49. Which months of the year do you have asthma? (Mark each applicable one)

a) January	Yes	No	g) July	Yes	No
b) February	Yes	No	h) August	Yes	No
c) March	Yes	No	i) September	Yes	No
d) April	Yes	No	j) October	Yes	No
e) May	Yes	No	k) November	Yes	No
f) June	Yes	No	l) December	Yes	No

CHEST COUGH

50. Do you cough when you wake up in the morning? (Mark one)

a) No - **go to question 56**	1
b) Yes, the previous 3 months	2
c) Yes, longer than the previous 3 months	3
d) Yes, the previous year	4
e) Yes, longer than the previous year	5

51. When do you cough mostly? (Mark one)

a) During the day	1
b) During the night	2
c) During the day and the night	3
d) Only when I wake up/go to bed	4

PHLEGM

52. Do you usually have phlegm on the chest? (Mark one)

a) No - **go to question 58**	1
b) Yes, with colds	2
c) Yes, without colds	3
d) Yes, with and without colds	4

f) Other, Specify ____________________________

53. If "Yes", has this phlegm been present for longer than 3 months of the year?

a) No	1
b) Yes, previous year	2
c) Yes, previous and other years	3

WHEEZE OF THE CHEST

54. Does your chest sound wheezy or whistling when you inhale?

a)	Yes	1
b)	No	2

If NO, go to question 66.

55. Does this wheeze of the chest occur with colds?

a) No	1
b) Yes, previous year	2
c) Yes, previous and other years	3

56. Does this occasionally happen other than with colds?

a) No	1
b) Yes, previous year	2
c) Yes, previous and other years	3

57. When does your chest wheeze mostly, when you **don't** have a cold?

a) During the day	1
b) During the night	2
c) During the day and the night	3

58. During which month(s) of the year do you usually have an episode of wheezing? (Mark each applicable one)

a) January	Yes	No	h) August	Yes	No
b) February	Yes	No	i) September	Yes	No
c) March	Yes	No	j) October	Yes	No
d) April	Yes	No	k) November	Yes	No
e) May	Yes	No	l) December	Yes	No
f) June	Yes	No	m) Unknown	Yes	No
g) July	Yes	No			

59. Have you had attacks of shortness of breath when your chest wheezes?

a) No	1
b) Yes, previous year	2
c) Yes, previous and other years	3

60. Do you ever get attacks of wheezing during or after you have been exercising?

a)	Yes	1
b)	No	2

61. Have you ever taken any medication for these attacks?

a)	Yes	1
b)	No	2

62. Have you ever been hospitalized for **<u>respiratory</u>** ailments?

a)	Yes	1
b)	No	2

If yes, please provide the following information:

c) How many times? _______

d) If possible, specify month and year of **each** admittance:

i) ______________________

ii) ______________________

iii) ______________________

iv) ______________________

v) ______________________

vi) ______________________

e) List reasons for admittance:

____

OTHER ILLNESSES AND CONDITIONS

63. Are you currently using any medication prescribed by a doctor?

a)	Yes	1
b)	No	2

If yes, please specify ___________________

64. Within the past **year**, have you had any of the following? (Mark Yes **or** No for each one)

a) Bronchitis	Yes	No
b) Pneumonia	Yes	No
c) Running nose	Yes	No
d) Earache	Yes	No
e) Hay fever	Yes	No
f) Sinusitis	Yes	No
g) Asthma	Yes	No

65. Have you had any other major illnesses or accidents that kept you home or prevented you from participating in any activities during the past year?

a)	Yes	1
b)	No	2

If yes, please specify ___________________

66. Have you **ever** had any of the following? (Mark Yes **or** No for each one)

a)	Learning disability	Yes	No
b)	Hyperactivity	Yes	No
c)	Hepatitis	Yes	No
d)	Cancer	Yes	No
e)	Gastro-intestinal diseases	Yes	No
f)	Heart disease	Yes	No
g)	High blood pressure	Yes	No
h)	Stroke	Yes	No
i)	High cholesterol	Yes	No
j)	Diabetes	Yes	No
k)	Painful joints (arthritis/gout)	Yes	No

67. During the past **12 months** how often have you been bothered by any of the following?

a) Eye problems _________ times
b) Nose problems _________ times
c) Ear problems _________ times
d) Throat problems _________ times
e) Chest/lung problems _________ times
f) Skin problems _________ times
g) Stomach/abdomen problems _________ time

68. Has anyone in your immediate family ever had any of the following? (Mark Yes **or** No for each one)

a) High blood pressure	Yes	No
b) Stroke	Yes	No
c) Heart attack/angina/chest pain while exercising	Yes	No

d) If yes in c above, was it before age 50? | Yes | No |

69. Do you consider yourself as?

a) Overweight	1
b) Underweight	2
c) Normal weight	3
d) Unknown	4

INFORMATION ON OCCUPATION

70. How many years of schooling or other education have you completed?

a) Did not complete std. 8 (Grade 10)	1
b) Completed std. 8 (Grade 10)	2
c) Completed std. 10 (Grade 12)	3
d) Completed Degree Diploma	4
e) Completed Post-graduate qualifications	5

71. What is your current employment status?

a) Employed full time	1	d) Student	4
b) Employed part time	2	e) Unemployed	5
c) Self-employed	3	f) Other	6

72. What is your current occupation or job?

a) Job or occupation (please be specific)

____

b) Type of business or industry

____

c) Number of years worked at this job

____

73. Have you ever worked in a dusty environment for more than a year?

a)	Yes	1
b)	No	2

c) If yes, please specify

____

74. Have you ever been exposed to gases and/or chemicals in the work place?

a)	Yes	1
b)	No	2

c) If yes, please specify

____

PERSONAL VIEWS

75. In your opinion, what factor(s) has the biggest influence on your respiratory health status?

____

____

76. Do you perceive the air pollution in the KZN as serious?

a) Yes	1	c) Not critical	3
b) No	2	d) Unknown	4

77. If you do not live in......................... any more, do you perceive air pollution levels in the area where you currently live as serious?

a) Yes	1	c) Not critical	3
b) No	2	d) Unknown	4

78. What do you consider the most important source of air pollution in your area? (Mark one)

a) Motor vehicles	1
b) Industries and mines	2
c) Cigarette smoke	3
d) Open fires (from areas without electricity)	4
e) Other (Specify) ____________________	

79. Have you noticed unusual odours in your neighbourhood?

a) Yes	1
b) No	2

80. If "Yes" for how long have you noticed these odours? (Complete only one)

a)			Years
b)			Months
c)			Days

81. Do you feel that these odours are affecting your health?

a) Yes	1
b) No	2
c) Unknown	3

82. If "Yes" how severely do you feel these odours are affecting your health?

a) A great deal	1	c) Very little	3
b) Fairly	2	d) Unknown	4

[1] This questionnaire was based on several other questionnaires used in epidemiological studies on respiratory diseases. These are the ATS-DLD-78-A questionnaire, used for respondents of 13 or more years of age (Ferris, B.G. *Epidemiology Standardization Project. Contract No 1-HR-5-3028. Report HR-53028-F.* Division of Lung Diseases, National Heart, Lung and Blood Institute: city, state abbrev, USA, 1978), the Canadian Air Quality and Health Study questionnaire (NHW/HPB-190-03040), the Harvard School of Public Health's

Children's Health Study Questionnaire (NHW/HPB-190-03210) and the Vaal Triangle Air Pollution and Health Study (VAPS) questionnaire. All of these questionnaires have been extensively tested and reviewed by a large body of experts.
